# Targeted indoor residual insecticide applications shift *Aedes aegypti* age structure and arbovirus transmission potential

**DOI:** 10.1038/s41598-023-48620-5

**Published:** 2023-12-02

**Authors:** Oscar David Kirstein, Carlos Culquichicon, Azael Che-Mendoza, Juan Navarrete-Carballo, Joyce Wang, Wilberth Bibiano-Marin, Gabriela Gonzalez-Olvera, Guadalupe Ayora-Talavera, James Earnest, Henry Puerta-Guardo, Norma Pavia-Ruz, Fabian Correa-Morales, Anuar Medina-Barreiro, Pablo Manrique-Saide, Gonzalo M. Vazquez-Prokopec

**Affiliations:** 1 Department of Environmental Sciences, Emory University, 400 Dowman Dr. 5Th Floor, Suite E530, Atlanta, GA USA; 2 Unidad Colaborativa de Bioensayos Entomológicos, Campus de Ciencias Biológicas y Agropecuarias, Universidad Autónoma de Yucatán, Mérida, Yucatán Mexico; 3 Laboratorio de Virología, Centro de Investigaciones Regionales “Dr. Hideyo Noguchi”, Universidad Autónoma de Yucatán, Mérida, Yucatán Mexico; 4 Laboratorio de Hematología, Centro de Investigaciones Regionales “Dr. Hideyo Noguchi”, Universidad Autónoma de Yucatán, Mérida, Yucatán Mexico; 5 CENAPRECE, Ministerio de Salud de Mexico, Ciudad de Mexico, Mexico

**Keywords:** Ecology, Population dynamics, Translational research

## Abstract

While residual insecticide applications have the potential to decrease pathogen transmission by reducing the density of vectors and shifting the age structure of the adult mosquito population towards younger stages of development, this double entomological impact has not been documented for *Aedes aegypti*. *Aedes* collected from households enrolled in a cluster-randomized trial evaluating the epidemiological impact of targeted indoor residual spraying (TIRS) in Merida, Mexico, were dissected and their age structure characterized by the Polovodova combined with Christopher’s ovariole growth methods. In total, 813 females were dissected to characterize age structure at 1, 3, 6, and 9 months post-TIRS. Significant differences in the proportion of nulliparous *Ae. aegypti* females between the treatment groups was found at one-month post-TIRS (control: 35% vs. intervention: 59%), three months (20% vs. 49%) but not at six or nine months post-TIRS. TIRS significantly shiftted *Ae. aegypti* age structure towards younger stages and led to a non-linear reduction in survivorship compared to the control arm. Reduced survivorship also reduced the number of arbovirus transmitting females (those who survived the extrinsic incubation period). Our findings provide strong evidence of the full entomological impact of TIRS, with important implications for quantifying the epidemiological impact of vector control methods.

## Introduction

The global spread of *Aedes*-borne viruses (ABVs) such as dengue (DENV), Zika (ZIKV), and chikungunya (CHIKV) is a significant public health issue that contributes to the growing global burden of urban illness^1^. The control of ABVs, which are primarily vectored by *Aedes aegypti*, presents significant difficulties because of the lack of effective vaccines and therapeutics, limited budgets and coverage of vector control programs, and low effectiveness of existing vector control tools (primarily focused on outdoor fogging and larval control), and the occurrence of insecticide resistance^2–4^. The significant global health emergency imposed by the recent Zika epidemic has catalyzed the development and evaluation of novel approaches for vector control and is advancing the field of urban *Aedes* vector control (3, 4).

The application of residual insecticides on lower wall and surfaces inside homes where *Ae. aegypti* mosquitoes primarily rest, known as targeted indoor residual spraying (TIRS), constitutes an improvement from classic IRS that can be deployed within urban areas at a lower insecticide consumption, time and cost per home^5,6^. Moreover, while less insecticide and time are used per home compared to IRS, TIRS does not translate in any reduction in insecticide efficacy or residuality^5^. TIRS was associated with a ~ 90% reduction in DENV transmission during an outbreak in Cairns, Australia^7^. In Mexico, applying TIRS preventively (prior to the typial ABV transmission season) reduced *Ae. aegypti* density during the ABV transmission season to the same levels as during the low dengue transmission season (over a 7-month period)^6^. The potential for TIRS to be deployed at scale depends on its scalability and the magnitude of its epidemiological impact. Because TIRS only impacts the indoor adult mosquito population, leaving larval habitats unaltered, we hypothesize that in addition to reducing *Ae. aegypti* density, TIRS may have a significant effect in shifting the mosquito population towards a younger age structure. This hypothesized double impact (reducing population density and shifting age structure) could explain the high efficacy of TIRS in reducing DENV transmission in Australia even in the absence of full insecticide coverage^7,8^.

Theory predicts that interventions altering the mosquito population age structure can lead to significant reductions in pathogen transmission even in the presence of mosquitoes^9–11^. The short duration of ABV viremia in human hosts, in comparison to the longevity of infected mosquitoes in the field, provide insight into the mechanism by which changes in age structure may impact transmission. Shifting the population towards younger ages may lead to a higher proportion of mosquitoes not surviving the extrinsic incubation period, which will translate into important reductions in pathogen transmission^11^. Several factors may lead to a shift in mosquito age structure. Differential mortality in older age groups can influence age structure towards younger mosquitoes (this, indeed, was the mechanism behind the *wMelpop* strain of *Wolbachia*^12^). Vector control methods that target adults, but not larval habitats, could also shift vector population age structure as long as the rate of mortality in adults exceeds the rate of emergence from larval habitats (i.e., long duration of insecticidal effect or very high mortality by the method are able to skew the population structure towards younger mosquitoes). Quantifying the impact of vector control on mosquito age is also challenged by difficulties in age grading mosquitoes within an operational field setting^13^.

Johnson et al.^13^ reviewed different methodologies for determining mosquito age and emphasized the importance of developing guidelines for evaluating vector control methods, highlighting the need to use entomological-based standards for predicting epidemiological outcomes. Accurately grading mosquito age and determining survival rates is a crucial technique for understanding mosquito biology, behavior, and population dynamics, as well as more accurately modeling and predicting the impact of interventions^13^. Novel methods for age grading such as near-infrarred spectroscopy have shown great accuracy in laboratory conditions, but have not yet been able to accurately and reliably predict the age of mosquitoes in natural environments^14^. This challenge has lead scientists to continue relying on on ovary dissections to quantify age structure in natural populations^15,16^. Very few *Ae. aegypti* interventions have been evaluated on their impact on age structure, all of them using parity rate as a proxy. In Iquitos, Peru, Morrison et al.^17^ found no significant effect of transfluthrin spatial repellents on *Ae. aegypti* parity rate whereas Gunning et al.^18^ found that conducting indoor space spraying lead to an immediate doubling of the proportion of nulliparous females in treatment areas versus the baseline conditions. Unfortunately, no age-specific estimates beyond parity have been used to quantify the impact of any intervention controlling *Ae. aegypti*, limiting the understanding of how observed entomological impacts of an intervention can translate into epidemiological outcomes.

The TIRS trial is a NIH-funded cluster randomized controlled trial quantifying the epidemiological impact of TIRS on ABVs in the city of Merida, Mexico. The primary enpoint of the trial quantifies the impact of the intervention on laboratory confirmed ABV illness detected in a cohort of 4600 children aged 2–15 at enrollment and living within 50 clusters measuring approximately 5 × 5 city blocks each^19^. Secondary endpoints include ABV infection (measured by annual serological surveys) in the human population as well as the impact of TIRS on *Ae. aegypti* density. As part of the trial, and in order to generate information to calibrate a mathematical model used to project TIRS efficacy^20^, we conducted a study quantifying the joint impact of TIRS on the density of female *Ae. aegypti* mosquitoes and the age structure of the mosquito population.

## Material and methods

### Study site

This research took place in Merida, which is the largest city in southeast Mexico and also the capital of the Yucatan state. Merida has a population density of 3889 people/km^2^ and a total population of ~ 900000, as per the 2020 census. The climate in Merida is classified as tropical savanna according to the Köppen climate classification system. The city experiences high temperatures throughout the year, with an average annual high of 33.5 °C. Temperatures can exceed 38 °C during the hottest months of May and June, while the lowest temperatures range from 17.2 °C to 21.7 °C between January and February. The rainy season lasts from June through October, with an annual cumulative precipitation of 1000 mm (22). Arboviral diseases are endemic in Merida due to favorable environmental conditions^21^. Dengue has been persistently transmitted in the area since 1979, while Chikungunya and Zika have been co-circulated since 2015^22,23^. The peak transmission season for arboviral diseases is from July to November, although cases can occur throughout the year^24^. The incidence of these infections follows the seasonality of *Aa. aegypti*, which are most abundant between August and October.

### Study design and data collection

This study was conducted on a subset of houses found within the 50 neighborhood clusters that were part of the TIRS trial between June 23rd 2021 and April 2nd 2022. Randomization for the TIRS trial was conducted using covariate-constrained randomization, as described in^25^. The study consisted of a TIRS treatment phase (lasting from late April to beginning of June 2021) conducted only in the 25 clusters that were part of the treatment arm, and an entomological surveillance phase conducted at 1, 3, 6, and 9 months post-TIRS in a subset of 1500 houses distributed 1:1 between treatment and control arms. The organophosphate insecticide pirimiphos-methyl (Actellic 300CS®) was applied following WHO/PAHO protocols^26^. In both clusters, vector control actions (truck-mounted ULV spraying using malathion) could be performed in response to symptomatic ABV cases reported to Yucatan’s Ministry of Health (YMOH). Throughout 2021, 23 control and 22 TIRS clusters received ULV spraying with malathion at an average of 1.9 times per year. The very low frequency of ULV sprays and balanced distribution between treatment and control arms led us to assume that the effect of this sporadic source of control on *Ae. aegypti* age structure would be negligible.

During the entomological surveillance phase, mosquitoes were collected using Prokopack aspirators^27^ from a subset of all houses enrolled in the study to match our estimated power calculations (see below). Adult indoor resting mosquitoes were collected for 10 min in each house using Prokopack aspirators by teams of 2 trained field collectors. No personal pesticide or repellent was used. Collected mosquitoes were transported alive to the Autonomous University of Yucatán's Collaboration Unit for Entomological Bioassays (UCBE-UADY) for sexing, species identification and dissections and then preserved in RNALater (Thermo Fisher Scientific, Waltham, MA, USA) with 1.5µl Tween® 20 (Sigma-Aldrich Co.) at − 80 °C for further molecular investigations. World Health Organization cone bioassays were conducted monthly up to 8 months post-intervention on ten houses receiving treatment and ten control houses using laboratory-reared susceptible Ae. aegypti females (Rockefeller strain) and estimating 24h mortality.

### Sample size calculation

We aimed to detect a 25% or greater difference in the proportion of nulliparous female *Ae. aegypti* between the intervention and control clusters. To achieve this, we sampled six female mosquitoes per cluster in each of the 50 clusters, totaling 300 females per survey date, assuming a 5% significance level and 80% power. With this sample size, we would be able to quantify an Odd Ratio (OD) of 0.21 or lower for binary outcomes, and Incidence Rate Ratios (IRR) of 0.29 or lower for count outcomes (Table S1). All females collected in houses in which dissections were performed were included in the statistical analysis to assess the efficacy of the intervention.

### Physiological age determination

**Figure 1:**
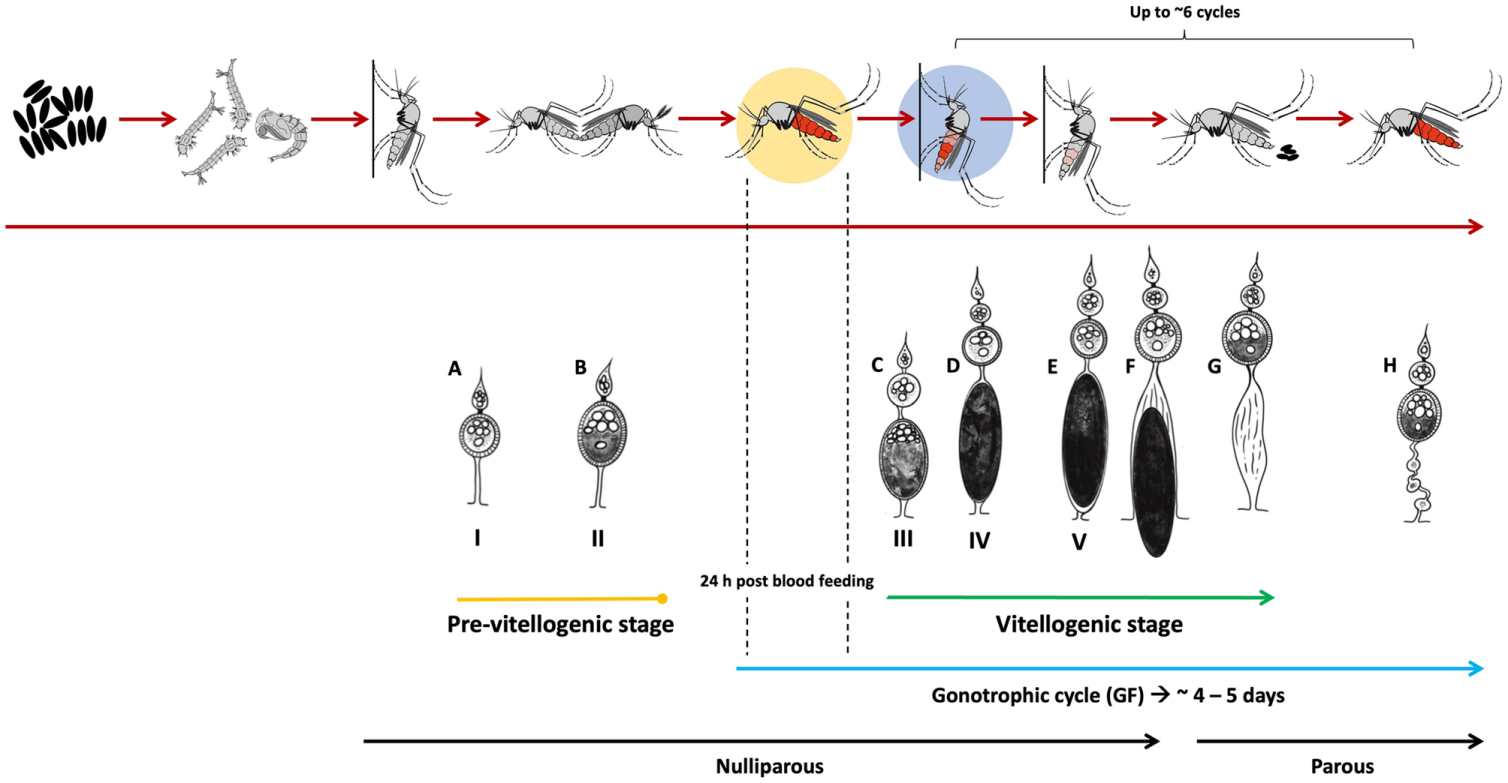
Life cycle of *Aedes aegypti*, emphasizing the crucial stages of the gonotrophic cycle. This cycle holds significant importance in comprehending mosquito reproduction and its involvement in pathogen transmission. Upon completion of their pupal stage, mature mosquitoes emerge and commence their adult phase. The gonotrophic cycle is initiated when female mosquitoes obtain their initial blood meal with enough volume to initiate egg development (*Ae. aegypti* may feed multiple times until such volume is reached). The process of follicular development can be divided into several stages (Christopher’s phases). Following the initial blood meal, the ovaries undergo a series of developmental changes in preparation for egg production, culminating in the oviposition of eggs.

To determine the physiological age of female *Ae. aegypti*, we relied on three primary measures. Firstly, we used Polovodova's method to examine changes in the morphology of the reproductive system and identify "follicular pedicel relics or dilatations".^15^. Follicular dilatations are scars that develop every time an egg is shed from an ovariole. The number of pedicel dilations reflects the number of completed gonotrophic cycles, enabling us to not only count the number of cycles but also classify females into nulliparous (young females who have not laid eggs) and parous or multiparous females (older females who have already taken a blood meal and laid eggs) (Fig. 1). Secondly, we examined the follicular stage of ovarioles during oogenesis, which reflects the maturation grade of an egg follicle within an individual ovariole. We followed Christophers' five-stage classification of follicular development in female *Ae. aegypti*^16^, ranging from growth stages or previtellogenic stage (FI-FII) to germinative stages or vitellogenic stage (FIII-FV). The FI stage occurs solely in unfed nulliparous females and continues to grow until the FII stage if a female is mated but not fed. A blood meal initiates the third phase (FIII) and can complete the rest of the trophic phases (FIV) until reaching the elongated mature shape (FV). After oviposition, the saccular stage could be identified, where the ovariole stalk remains slightly swollen until the ovariole dilatations are defined (Fig. 1). Figure S1 shows examples of dissections indicating different dilation and follicular stages. Lastly, we classified blood-fed females by the degree of blood digestion using the Sella score before ovary dissections. We extended the Sella scale to include recent feeding, where bright red blood, regardless of volume, indicated blood feeding within 24 h of collection and was assigned a Sella score of 2. Typically, a female *Ae. aegypti* takes 2–3 days to complete a blood meal, while egg development from stage FI to stage FV takes 1.67 to 5 days after blood meal completion under ideal temperature conditions at 28.9°C^28^. Additionally, egg hatching takes three days on average under controlled conditions.

In order to obtain accurate measurements and prevent the growth of follicles in the ovarioles, female *Ae. aegypti* were immobilized by freezing and dissected within 8 h of being collected. Individual female mosquitoes were dissected in phosphate buffer saline (PBS) following the Polovodova method^15^. The ovaries were carefully removed, the ovarian sheath detached, and the ovarioles were separated using dissecting needles under a stereoscope magnified at 40x. Subsequently, the ovarioles were stained for 1 min with a Neutral Red solution, prepared daily in PBS. They were then mounted and observed without a coverslip under a compound microscope with Differential Interference Contrast (DIC) optics at 100 × and 200 × magnification. Dissections were conducted by two trained entomologists (CQ and JC), who conducted practices using laboratory-reared mosquitoes with known gonotrophic history prior to conducting this study.

### Measures for analysis

We evaluated the following entomological variables, including: 1) the density of female *Ae. aegypti* captured indoors (count variable); 2) the engorgement status (binary variable, unfed = 0, bloodfed = 1) and weight (continuous variable) of female *Ae. aegypti* using Sella's score; 3) Parity (binary, nulliparous = 0, parous = 1); and 4) the “physiological age” of female *Ae. aegypti* (ordinal categorical variable) (Fig. 1). In the absence of age-specific mortality (which is the case of insects), mosquito reproductive age structure (measured as the number of dilations in dissected females) can be used to estimate population-level survivorship curves^11^. In the context of our study, more dilations are indicative of more gonotrophic cycles, which are a primary index of reproductive age^11^. The shape of the line fitted to a plot with the reproductive age (number of dilations) in the x-axis and the natural logarithm of the number of females on each age class in the y-axis allows inferring whether mortality varies linearly or non-linearly with age^11^. We used maximum likelihood to fit different functions to the data and compared whether a non-linear fit predicted the data better than a linear fit using the Matlab Curve fitting module (Mathworks).

We estimated the transmissibility potential of each dissected female by monitoring the number of follicular dilatations and the follicular stage. For a female *Ae. aegypti* to acquire the ability to transmit arboviruses to a susceptible host, it requires an incubation period (EIP) of 8 to 12 days. During this period, the female mosquito undergoes two follicular dilatations (indicating two blood meals and two gonotrophic cycles) and advances to the FII follicular stage till the next blood intake. Consequently, we identified females with more than one dilatation and at or beyond the FII follicular stage as potential arbovirus transmitters (that could have survived the EIP) or non-transmitters.

### Statistical analysis

To detect differences between the intervention and control groups, we used Chi-square tests (*χ*^2^) for categorical variables and unconditional generalized linear mixed models using random-effects Poisson (count variables) and logistic (binary variables) models with cluster ID as the random intercept. Models were used to estimate incidence rate ratios (IRR) and odds ratios (OR), respectively, including 95% CI limits, as in^6^. TIRS efficacy was calculated as Efficacy = 1-IRR for count outcomes and Efficacy = 1-OR for binary outcomes^29^. The statistical analyses and data visualizations were carried out using Stata SE 17 and the R programming environment (https://www.r-project.org/), along with the packages 'stats,' 'lme4', and 'ggplot2'^30^.

### Ethical considerations

This study was conducted in adherence to the TRIS trial protocol and all applicable rules and legislation. Prior to intervention and entomological survey visits, written informed consent was obtained from the household owner. The TIRS study protocol, informed consent forms, and supporting documents were approved by the Institutional Review Boards of Emory University (IRB00108666) and Universidad Autónoma de Yucatán (UADY) (CEI-05-2020). Additionally, the trial protocol was registered on clinicaltrials.gov (NCT04343521, registered on 13/04/2020).

## Results

### Impact of TIRS on infestation and density

TIRS was applied to a total of 8241 houses and took an average of 10.6 (SD ± 4.6) minutes per house. Intervention coverage (measured as the % of enrolled houses in a cluster that received the intervention) averaged 0.67 (95% CI 58.3–63.3). WHO cone bioassays confirmed the residual efficacy of Actellic 300CS to be about 5–6 months (based on 80% mortality threshold) (Fig. S2). Entomological surveys led to the collection of a total of 6503 *Ae. aegypti* (4059 from control and 2444 from TIRS clusters) of which 44% (N = 2920) were females. Dissections were performed on 813 females collected from 564 houses throughout all the surveys (Table 1).

**Table 1:** Cluster characteristics and entomological characteristics by households.

Characteristics	Total			
	Control		Intervention	
	N	%	N	%
Number of clusters	25	50.0	25	50.0
Number of households	1500	50.0	1500	50.0
Positive cluster				
Female Ae. aegypti*	1793	(17.9 ± 16.4)	1127	(11.3 ± 11.5)
Fed female Ae. aegypti*	1433	(14.3 ± 13)	981	(9.8 ± 10.2)
Unfed female Ae. aegypti*	360	(3.6 ± 5.1)	146	(1.5 ± 1.8)
Male Ae. aegypti*	2266	(22.7 ± 27)	1317	(13.2 ± 15.7)

*Mean and SD.

**Figure 2:**
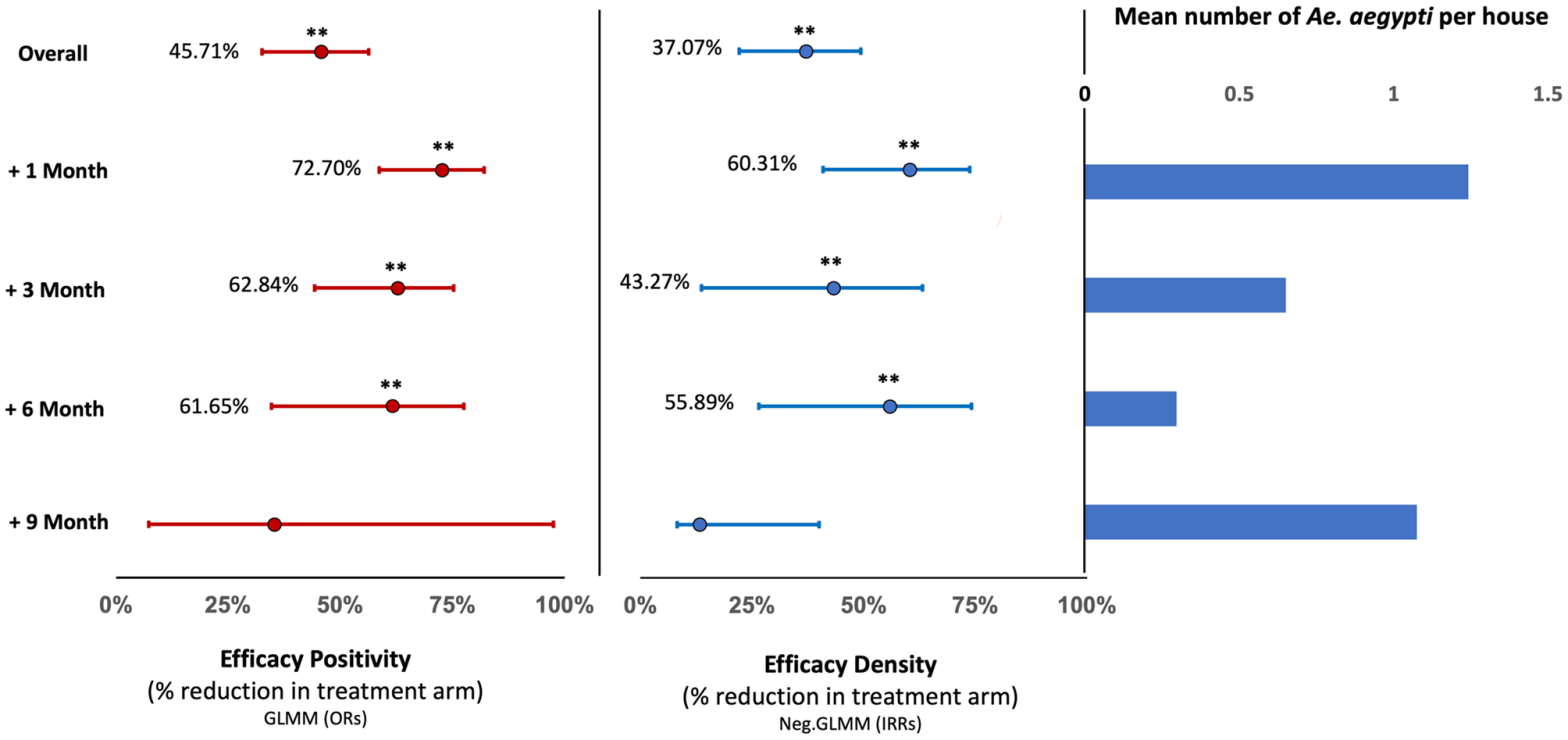
Estimated intervention effectiveness. Expressed as the proportional reduction compared to the control arm in reducing *Ae. aegypti* Positivity (**A**) and Density (**B**) per house and the predicted 95% CI (error bars) for each of these estimates. The overall efficacy was determined based on the total effect (during the nine months following the intervention). The mean number of female *Ae. aegypti* per house in the control clusters is depicted in (**C**). *Significant effectiveness relative to the control (p < 0.001).

TIRS significantly reduced the presence of *Ae. aegypti* per house up to 6 months post spraying, with intervention efficacies ranging from 73 to 61% within that period (Fig. 2). At nine months post-TIRS, no significant reduction in *Ae. aegypti* presence was measured (Fig. 2). Similarly, Ae. aegypti density was reduced significantly up to 6 months and ranged from 60 to 56% and was not significantly impacted at 9 months post-TIRS (Fig. 2).

### Impact of TIRS on age structure

**Figure 3:**
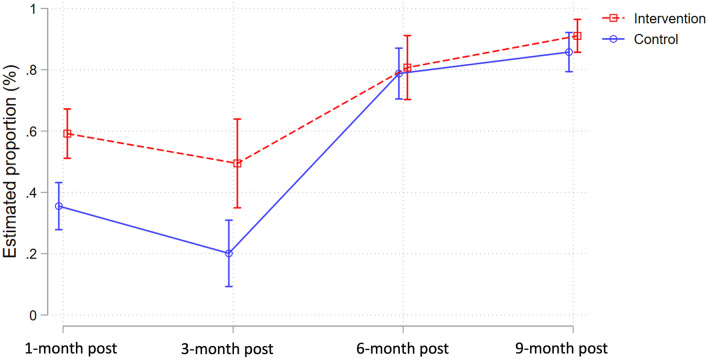
Proportion of nulliparous females over time in TIRS and control arms, with 95% CI estimates expressed as error bars.

**Figure 4:**
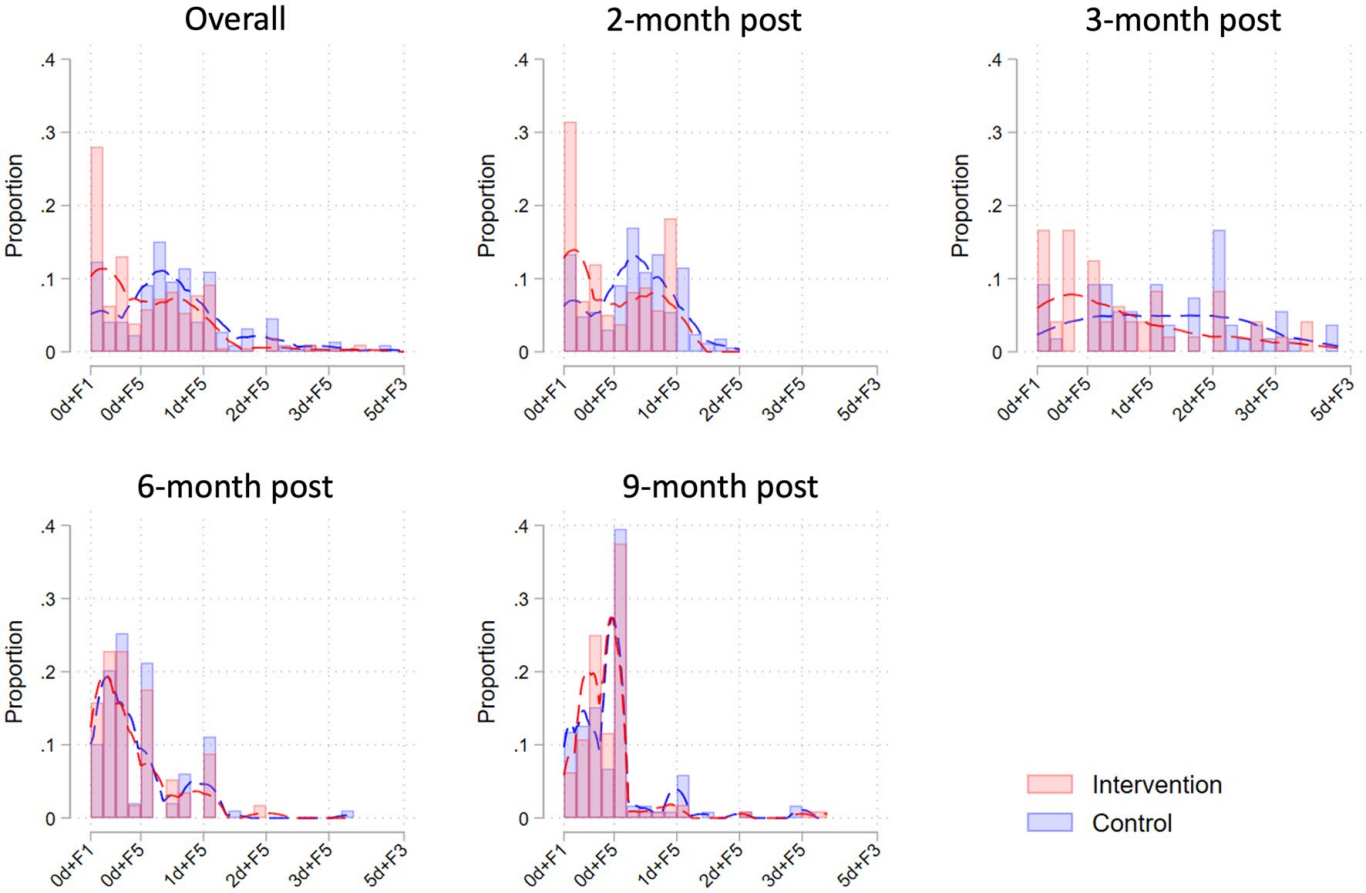
*Aedes aegypti* age structure, determined by quantifying the frequency of each physiological age class, determined by counting the number of dilations + the follicular stage (values in the x-axis are formatted accordingly, #d + F#). Lines indicate non-linear smoothing to characterize overall trendsin the data.

**Figure 5:**
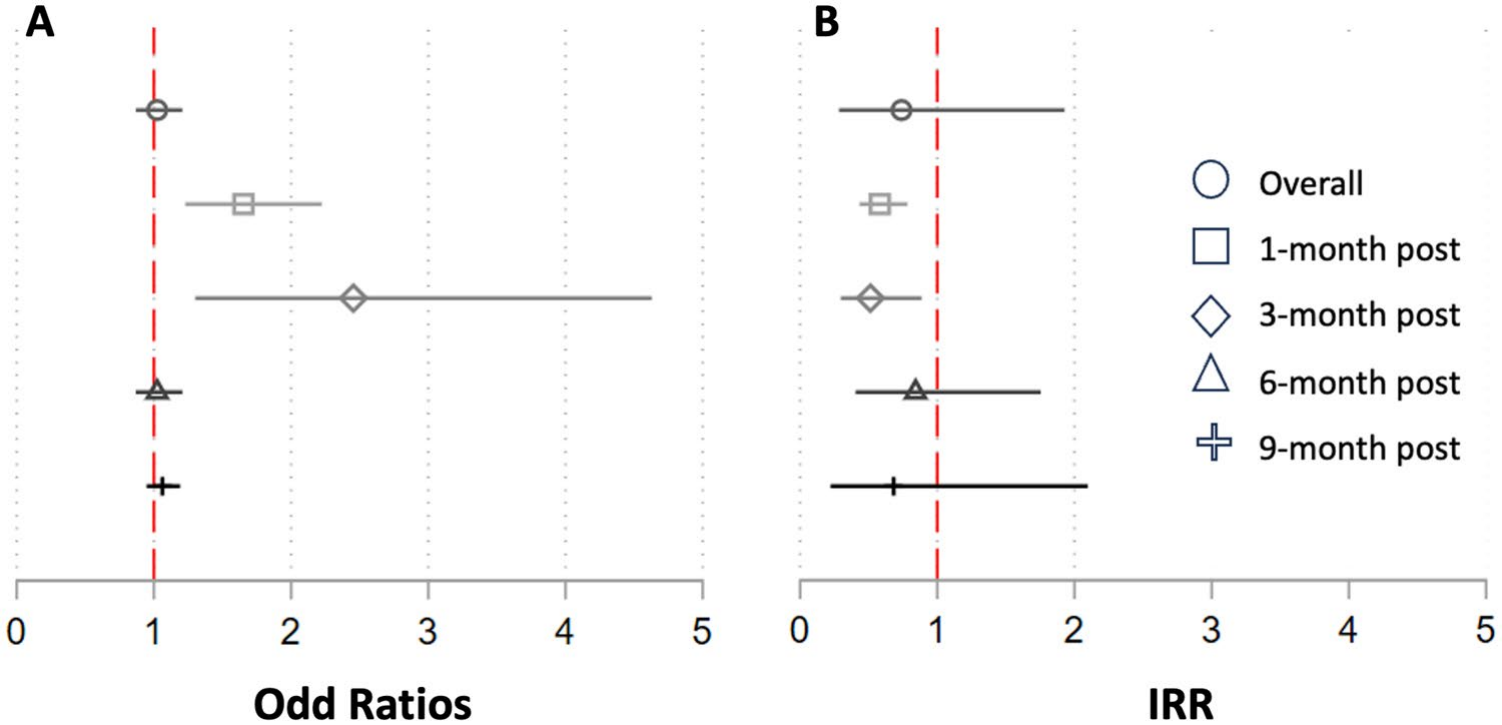
Results from generalized linear mixed models (GLMMs) applied on the parity (binomial link function) and the number of dilations (Poisson link function) and expressed as odds ratios or incidence risk ratios (IRR), respectively. The values indicate the unit value change in each metric in the TIRS arm, compared to the control arm (set as reference).

The proportion of nulliparous *Ae. aegypti* was significantly increased in the TIRS arm, compared to the control at one month (35.8% vs 59.1%, respectively; *χ*^2^ p = <0.001] and three months (20.4% Vs 50.0%; *χ*^2^ p = <0.001) post-TIRS (Table 2; Fig. 3). At six and nine months post-TIRS, however, there was no statistically significant difference in the proportion of nulliparous *Ae. aegypti* between trial arms (Table 2; Fig. 3). Similarly to the parity rate, the median number of ovariole dilations was significantly lower among the group one and three months after the intervention (p < 0.001) but comparable at six and nine months (p > 0.05) (Table 2). Parous female *Ae. aegypti* mosquitoes in the intervention group had fewer follicular dilations and were in earlier stages of follicular development (FI-FV) at one and three months post-intervention compared to the control arm, but there was no difference in the follow-up surveys (Fig. 4). Results from binomial GLMMs show that the proportion of nulliparous females was significantly increased in the treatment arm compared to the control arm at 1 and 3 months post-TIRS, but not thereafter (Fig. 5A; Table S2). Similarly, Poisson GLMMs showed a significant reduction in the number of dilations in the TIRS arm compared to the control arm at 1 and 3 months post-TIRS (Fig. 5B; Table S2).

**Table 2:** Descriptive and bivariate statistics of dissected female *Aedes aegypti.*

Characteristics	Total					2021—Post 1					2021—Post 3					2021—Post 6					2022—baseline				
	Control		Intervention		P**	Control		TIRS		P**	Control		TIRS		P **	Control		TIRS		P **	Control		TIRS		P**
	N = 437	%	N = 376	%		N = 165	%	N = 159	%		N = 54	%	N = 48	%		N = 99	%	N = 57	%		N = 119	%	N = 112	%	
Parity					< 0.001					< 0.001					0.002					0.78					0.21
Nuliparous	250	57.2	266	70.7		59	35.8	94	59.1		11	20.4	24	50.0		78	78.8	46	80.7		102	85.7	102	91.1	
Multiparous	187	42.8	110	29.3		106	64.2	65	40.9		43	79.6	24	50.0		21	21.2	11	19.3		17	14.3	10	8.9	
Gravity					0.11					< 0.001					0.57					0.10					0.22
Gravid	182	41.6	141	37.5		40	24.2	34	21.4		31	57.4	21	43.8		44	44.4	26	45.6		67	56.3	60	53.6	
Half-gravid	96	22.0	94	25.0		46	27.9	50	31.4		12	22.2	13	27.1		18	18.2	5	8.8		20	16.8	26	23.2	
Blood fed	62	14.2	39	10.4		31	18.8	1	0.6		5	9.3	7	14.6		17	17.2	18	31.6		9	7.6	13	11.6	
Unfed	97	22.2	102	27.1		48	29.1	74	46.5		6	11.1	7	14.6		20	20.2	8	14.0		23	19.3	13	11.6	
Follicular development					0.04					0.12					0.39					0.79					0.10
FI	96	22.0	91	24.2		54	32.7	63	39.6		16	29.6	11	22.9		10	10.1	9	15.8		16	13.4	8	7.1	
FII	75	17.2	60	16.0		28	17.0	25	15.7		5	9.3	5	10.4		24	24.2	16	28.1		18	15.1	14	12.5	
FIII	93	21.3	87	23.1		34	20.6	28	17.6		9	16.7	15	31.3		31	31.3	15	26.3		19	16.0	29	25.9	
FIV	26	5.9	40	10.6		14	8.5	22	13.8		1	1.9	2	4.2		2	2.0	1	1.8		9	7.6	15	13.4	
FV	147	33.6	98	26.1		35	21.2	21	13.2		23	42.6	15	31.3		32	32.2	16	28.1		57	47.9	46	41.1	

*Median and range, Kruskall-Wallis test of hypothesis.

**Chi-2 test.

**Figure 6:**
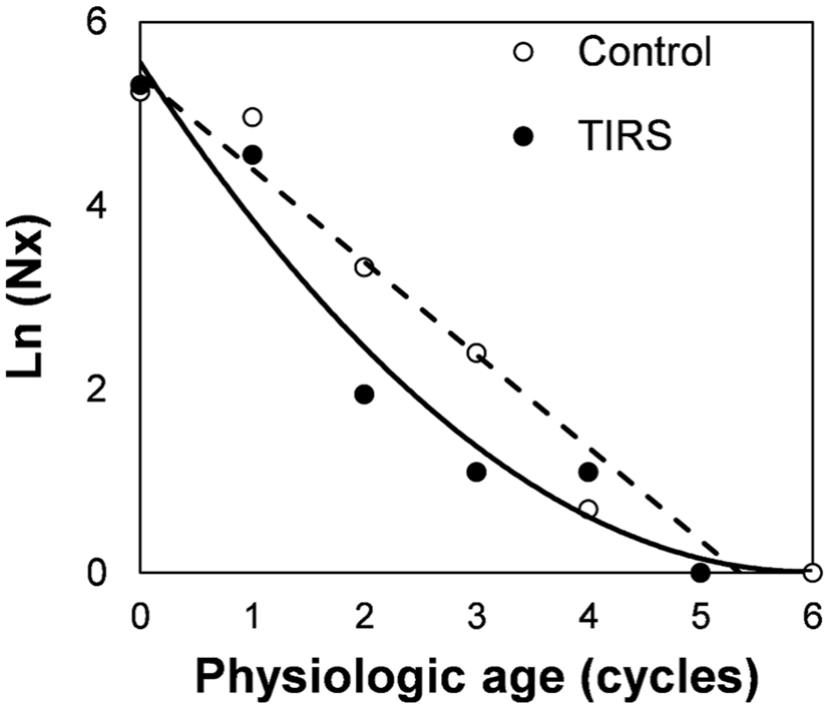
Survivorship of field collected *Ae. aegypti* females based on estimates of population age structure inferred by the number of ovary dilations as a proxy. Lines represent the fir to the data, which was split between control and TIRS arms.

We estimate adult survivorship in both trial arms and plotted it in Fig. 6. For the control arm, survivorship was significantly predicted by a linear trend $\left( {N_{x} = a + b*x} \right)$, with parameters (a = −1.010 [95% CI − 1.264 to − 0.757]) and b = 5.406 [4.493–6.319]) indicating constant mortality. In the TIRS arm, a non-linear age-dependent survivorship indicated a larger mortality at older mosquito ages $\left[ {N_{x} = a + b*exp\left( x \right)} \right]$, with parameter a = 5.730 [4.316–7.144] and b = −0.476 [− 0.689 to − 0.263]. Figure 6 also shows that the highest mortality in the TIRS compared to the control arm (evidenced by the largest separation between points) occurred at 2 dilations, which is consistent with females that have fed and laid eggs at least twice.

When splitting females by their physiological age with regards to their potential to transmit ABVs (ie. comparing the frequency of females with more than one dilation and FV follicular stage between arms), we find a significant reduction in the frecency of potential transmitter females in the TIRS arm compared to the control (19.4% vs 29.1%, respectively; *χ*^2^ P < 0.01; Table 3). Such reduction was significant at 1 and 3 months post-TIRS but not thereafter (Table 3). Interestingly, the most significant drop in the proportion of females with the potential to transmit ABVs occurred at 3 months post-TIRS (a 25.2% reduction; Table 3), coinciding with the peak density and transmission of ABVS in Merida.

**Table 3:** Female *Aedes aegypti* with potential for transmitting *Aedes*-borne viruses by treatment groups and entomological survey.

	< 1D + FV		> 1D + FV		P value*
	Non-transmitters		Transmitters		
	N = 613	%	N = 200	%	
Total					
Control	310	70.9	127	29.1	0.001
Intervention	303	80.6	73	19.4	
1 month post					
Control	105	63.6	60	36.4	0.02
Intervention	121	76.1	38	23.9	
3 months post					
Control	19	35.2	35	64.8	0.01
Intervention	29	60.4	19	39.6	
6 months post					
Control	80	80.8	19	19.2	0.41
Intervention	49	86.0	8	14.0	
9 months post					
Control	106	89.1	13	10.9	0.32
Intervention	104	92.9	8	7.1	

*Chi-square test.

## Discussion

Preventively applying TIRS had a significant population-level impact in *Ae. aegypti*. Within six months of TIRS application, mosquito density was significantly reduced, as also described previously^6^. More importantly, our study shows that within the first three months (the period of rapid mosquito population increase and ABV transmission in Merida) TIRS significantly increased the proportion of nulliparous *Aedes* mosquitoes and significantly reduced the number of ovariole dilations and the number of female mosquitoes old enough to potentially transmit ABVs. Our findings indicate that preventive residual interventions can exert a sustained impact on the mosquito population with important implications for ABV transmission.

Evaluation of vector control interventions against *Ae. aegypti* has long suffered from difficulties in associating a given entomological reduction to observed or expected case reductions^31^. Multiple factors may be responsible for this discordance, including the complex interaction between human mobility, inapparent ABV transmission and human immunity to ABVs^32^. Sampling factors, such as variable sensitivity of mosquito collection methods, are also influential^31^. A factor that is often ignored in this entomological-epidemiological assessment is the fact that changes in mosquito age structure due to an intervention can also influence ABV transmission even in the presence of mosquitoes^10,11,13^. This study shows the influence of TIRS on *Ae. aegypti* age structure beyond the quantification of parity. By quantifying a proxy of reproductive age, the number of ovarial dilations and stages of follicular development, we quantified how *Ae. aegypti* age structure changes due to TIRS and over time, revealing insights of the mechanism by which residual insecticides impact mosquito populations.

Residuality of insecticides is routinely assessed by bioassays that quantify the mortality of lab-reared mosquitoes exposed to a treated surface^33^. Such information is then used to determine how long the product is expected to last in field conditions^33^. We showed that applying TIRS with pirimiphos-methyl led to a significant reduction in *Ae. aegypti* density for up to 6 months (which is in agreement with previous findings from another randomized trial that found 7 months as the duration of population-level suppression^6^). Interestingly, surviving *Ae. aegypti* females were significantly younger in the TIRS compared to control arm. The non-linear survivorship curve we estimated for the TIRS arm (compared to a linear relationship of survivorship with age for the control) could be indicative that the intervention is significantly killing older females. While sampling bias towards younger adult mosquitoes could be other factor leading to a similar non-linear trend, Prokopack collections are not considered to be biased towards specific age groups as other methods such as gravid traps or ovitraps. This finding is critical to understand the mode of action of TIRS. Given larval habitats are not treated, TIRS does not impact adult mosquito recruitment directly. Therefore, reductions in population numbers may be driven by changes in the number of reproductive females. Analysis of the survivorship curve suggest that TIRS kills older females (2 + gonotrophic cycles) at a higher rate than natural mortality (natural mortality is shown as the linear trend observed in the survivorship of the control arm). We hypothesize that, because most human feeding occurs indoors, the majority of females collected in the TIRS area are either recently emerged or were able to ‘avoid’ insecticide contact during their first gonotrophic cycle, but not beyond it. Therefore, TIRS may lead to an age-dependent mortality curve that has important implications for ABV transmission beyond the reduction in mosquito density indoors. Findings like ours are rare, due to the difficulty in conducting dissections, but we evidence the importance of proper characterization of population age structure when evaluating a vector control tool^13^. For malaria vectors, for instance, a pivotal trial investigating the impact of insecticide impregnated nets led to the quantification of significant population-level impact of bednets on mosquito age structure and survioship before and after net introduction^34^.

An interesting finding of our study has been the lack of significant change in *Ae. aegypti* physiological age structure at 6 and 9 months post-TIRS. While at 9 months the lack of significance can be explained by the loss of residuality of pirimiphos-methyl^6^, this cannot explain the 6-month findings. Further exploration of age structure at this period provided useful insights. The 6-month post-TIRS survey occurred in December, which is a period of rapid weather changes in Merida that is associated with a strong seasonal reduction in *Ae. aegypti* numbers^35^. Comparing the age structure of *Ae. aegypti* between TIRS and control arms shows that the lack of statistical significance was due to a strong reduction in population physiological age in the control arm, not by an increase in physiological age in the TIRS arm; the latter would be expected if the insecticide lost its residuality and stopped killing adults in the presence of strong population recruitment. Therefore, our explanation for the 6-month phenomenon is that mosquito populations in the control arm died at a higher rate due to external factors (lower temperatures and increased drought during December) that made their population age structure shift towards ‘younger’ adults. Disentangling the influence of external forces of mortality leading to seasonality in mosquito populations is critical to identiy better windows for deployment of preventive mosquito control tools such as TIRS. It appears that the critical control phase occurs during the first 3–4 months post-application, when mosquito populations increase and their longevity is the highest. Unfortunately, the lack of similar studies conducted for *Ae. aegypti* limits comparing our findings with other settings or mosquito control tools.

Several considerations should be taken into account when interpreting our study. Firstly, the precision of our mixed effects model estimates relies on the number of clusters, which are the primary sample units. It is important to note that there was a reduction in the number of sampled clusters, which may affect the precision of our efficacy estimates, particularly in the six-month entomological assessment of the treatment group. However, we attribute this reduction to a decrease in available clusters with positive mosquito presence, resulting from an overall decrease in the household density of female *Ae. aegypti* observed at that time. While we powered the study to detect an OR of 0.2, we used χ^2^ tests to evaluate treatment effects on parity to account for the low sample size in TIRS arm due to the low number of mosquitoes found. Secondly, to gain a comprehensive understanding of arbovirus transmission dynamics, it would be valuable to measure the entomological inoculation rate (EIR), which evaluates the risk of exposure to infectious mosquitoes, typically represented as the number of infective mosquito bites received by an individual. For malaria vectors, pairing sporozoite rates and Anopheles age structure led to important insights of transmission potential and the impact of transmission-blocking interventions^36^. Unfortunately, detecting infection in *Ae. aegypti* is very difficult and dependent on the location, timing of recent transmission and sampling methodology^37^. Unfortunately, all mosquitoe collected in this study tested negative for ABVs by RT-PCR, limiting our ability to estimate EIR and transmission potential, as in^36^. Nevertheless, our results consistently demonstrate that the increased number of *Ae. aegypti* old enough to be potential transmitters contributes to an increased vectorial capacity, which adequately informs the potential for ABV transmission following TIRS application.

There is an ongoing debate regarding the identification of a reliable, reproducible, and accessible age-grading method^13^. Dissection methods such as the Polovodova method have limitations, including their reliance on trained personnel, potential subjectivity in interpretations by untrained technicians, and being labor-intensive, with each dissection taking an average of 25–30 min, which limits their use in large-scale studies. However, in our study, the dissection team was led by a senior entomologist who followed established dissection protocols (36, 37, 55, 56) and utilized *Ae. aegypti* reared under controlled conditions in bloodmeal chambers to validate findings from the field. Dissection methods for age-grading remain valuable due to their ease of implementation in low-income settings. Although modern age-grading methods, such as near- and mid-infrared spectroscopy, biochemical methods, and genetic profiling, may offer more precise estimations, they lack validation with empirical data and are currently inaccessible for vector control programs in low-income settings^13^.

Data on mosquito age structure are seldom considered explicitly in epidemiological models despite the acknowledgement by early medical entomologists of its relevance^10,11,36^. Even for TIRS, agent-based simulations conducted to project the epidemiological impact of the intervention have long assumed a constant mortality across all mosquito ages^20,38^. Our findings of changes in mosquito population physiological age structure, reproduction and survivorship due to TIRS provide important data to refine models and predictions of intervention impact that go beyond the consideration of reductions in mosquito density. The consequences of this theoretical exercise can be profound, as models can guide the design of interventions and the quantification of levels of coverage and entomological reduction required to exert a given epidemiological impact or can also identify potential synergies in the combination of interventions that target both adult density (e.g., TIRS, space spraying) and their population recruitment (e.g., larval control, autodissemination or *Wolbachia* for population suppression).

### Supplementary Information

Supplementary Information.

## Data Availability

All datasets used in this study are available to download from the following Mendeley Data link: Vazquez-Prokopec, Gonzalo (2023), “Targeted indoor residual insecticide applications shift *Aedes aegypti* age structure and arbovirus transmission potential”, Mendeley Data, V1. 10.17632/9tw3ngnjjj.1.
