# Supplementary material for: Targeted indoor residual insecticide applications shift Aedes aegypti age structure and arbovirus transmission potential
Source: Sci Rep. 2023 Dec 2;13:21271. doi: 10.1038/s41598-023-48620-5 (PMC10693548; doi:10.1038/s41598-023-48620-5)

**Supplementary Tables and Figures**

**Table S1.** Sample size calculation assuming α = 0.05, β = 0.8, and rho = 0.5

| **Difference in Proportions** | **Aedes per treatmet cluster (m1)** | **Aedes Control cluster (m2)** | **Treatmet clusters (K1)** | **Control clusters (k2)** | **N** | **OR**  **(m1/m2)** | **IRR (m1/m2)** |
| --- | --- | --- | --- | --- | --- | --- | --- |
| 25.97% | 4 | 4 | 25 | 25 | 200 | 0.198 | 0.278 |
| 25.34% | 5 | 5 |  |  | 250 | 0.203 | 0.283 |
| 24.91% | 6 | 6 |  |  | 300 | 0.207 | 0.286 |
| 24.37% | 8 | 8 |  |  | 400 | 0.212 | 0.291 |
| 24.04% | 10 | 10 |  |  | 500 | 0.215 | 0.294 |

**Figure S1**. Examples of ovariole dissections of field-collected *Ae. aegypti*, indicating (A) Ovariole without dilation (10x), (B) 0 dilatations +Follicular stage 5 (F5), (C) 4 dilations + follicular stage I (FI), and (D) 5 dilations + follicular stage I (FI).


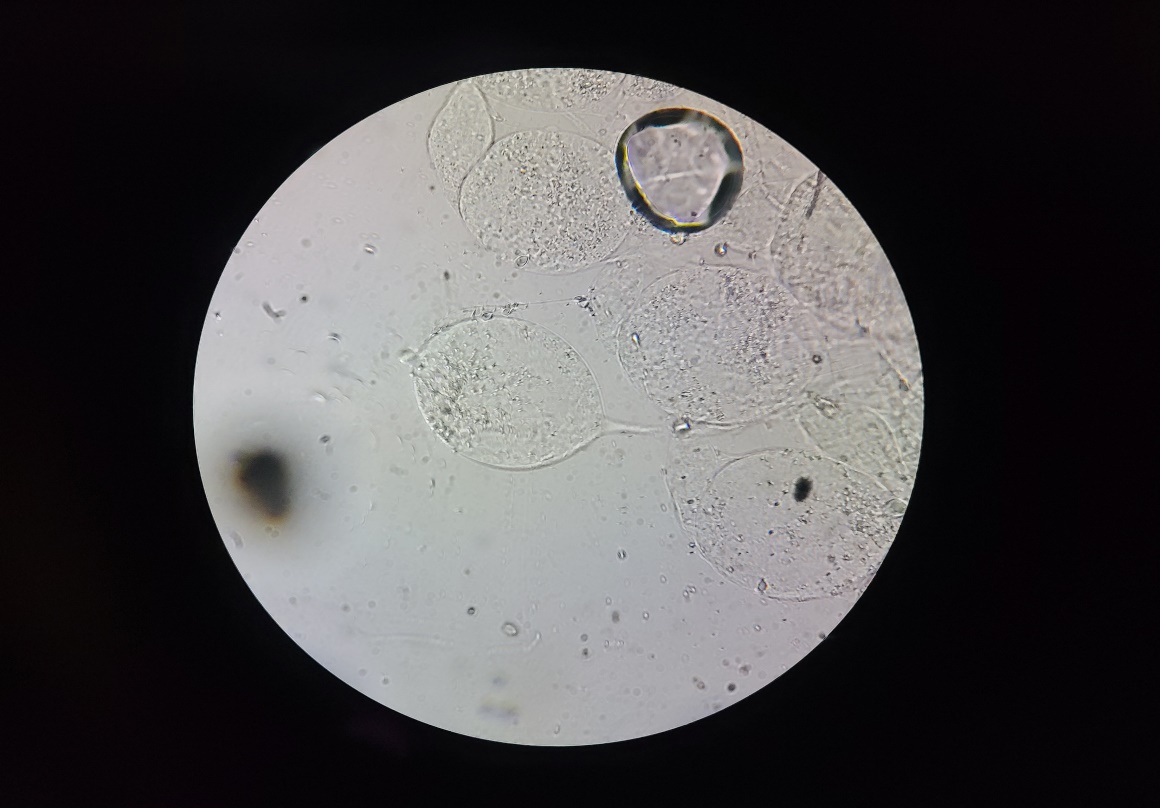

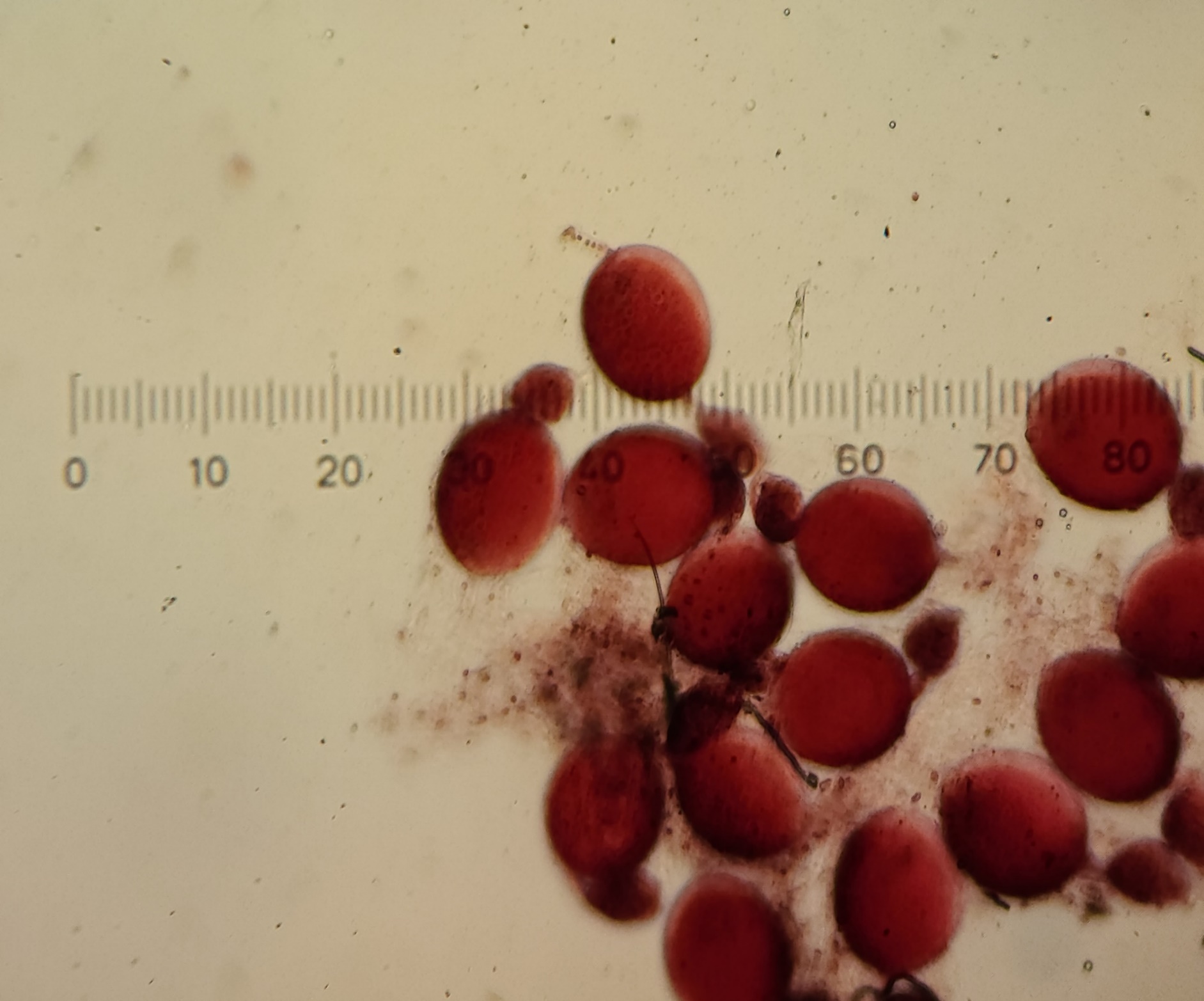

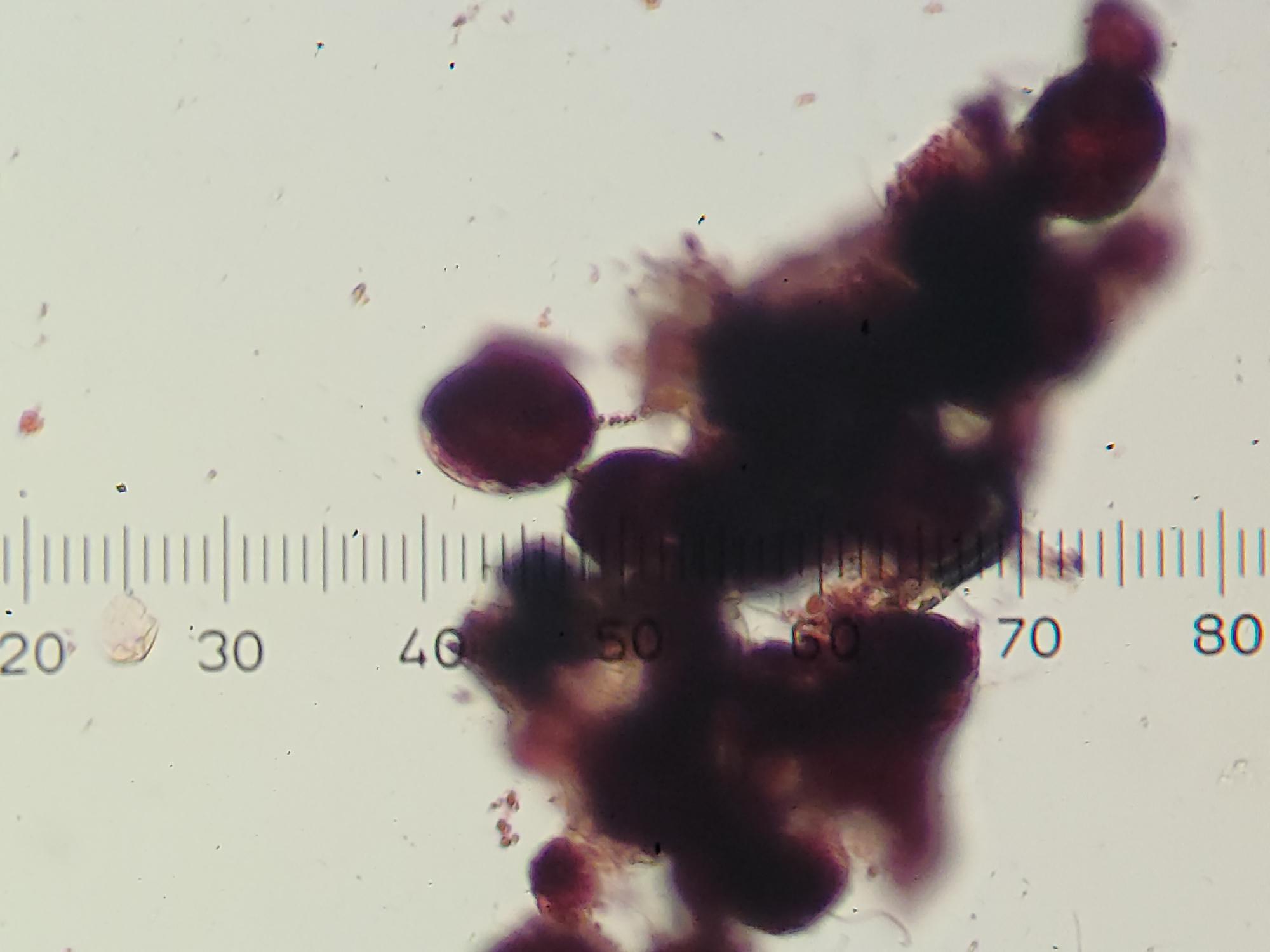


**A**

**C**

**D**


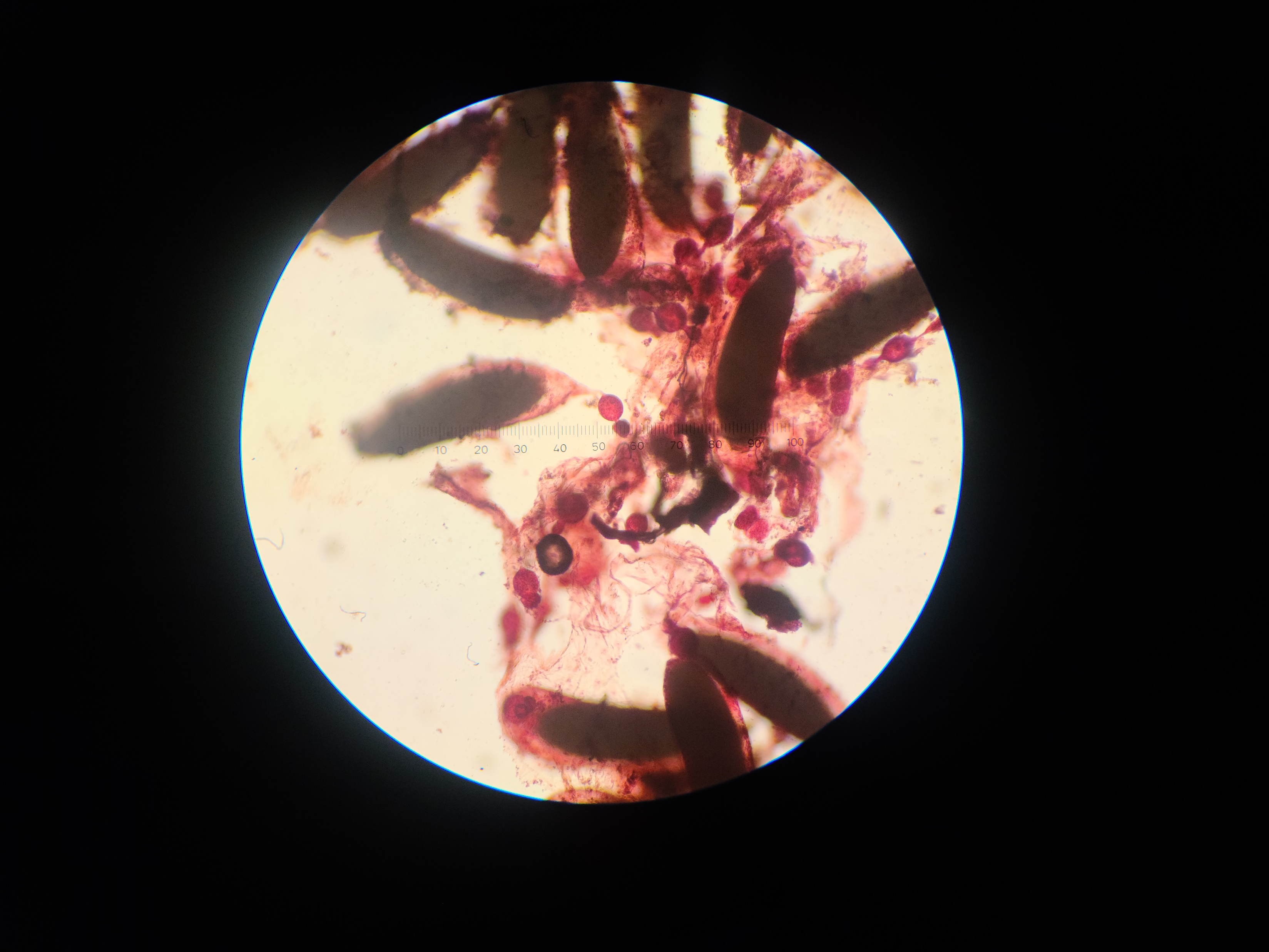


**B**

Figure S2. Results from WHO cone bioassays reporting mortality at 24h after the exposure of 100 mosquitoes for 30 min to walls treated and not treated with Actellic 300CS in Merida, Mexico.


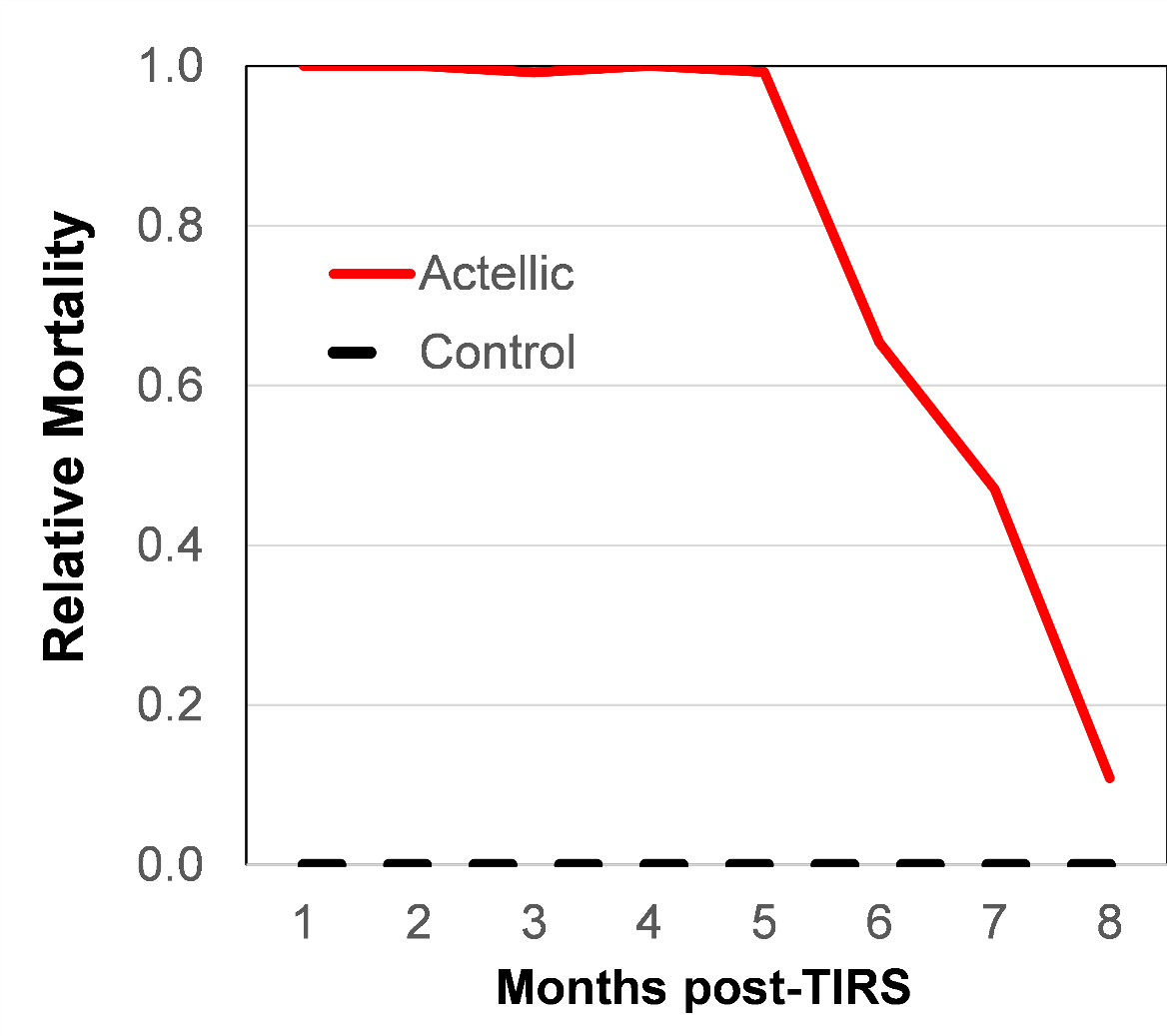

Supplement: Supplementary file 1 — Supplementary Information. [file 41598_2023_48620_MOESM1_ESM.docx]
